# SWCNT-Based Composite Films with High Mechanical Strength and Stretchability by Combining Inorganic-Blended Acrylic Emulsion for Various Thermoelectric Generators

**DOI:** 10.3390/nano15231817

**Published:** 2025-12-01

**Authors:** Yuto Nakazawa, Yoshiyuki Shinozaki, Hiroto Nakayama, Shuya Ochiai, Shugo Miyake, Masayuki Takashiri

**Affiliations:** 1 Department of Materials Science, Tokai University, Hiratsuka 259-1292, Kanagawa, Japan; 4cajm045@tokai.ac.jp (Y.N.); 5cajm022@tokai.ac.jp (Y.S.); 5cajm035@tokai.ac.jp (H.N.); 5cajm013@tokai.ac.jp (S.O.); 2 Department of Mechanical Engineering, Setsunan University, Neyagawa 572-8508, Osaka, Japan; shugo.miyake@setsunan.ac.jp

**Keywords:** stretchable, mechanical strength, SWCNT, composite films, acrylic emulsion, TEG, energy harvesting, IoT

## Abstract

Single-walled carbon nanotube (SWCNT) films are potential materials for thermoelectric generators (TEGs) owing to their flexibility and high thermoelectric performance near 300 K. However, they inherently exhibit low mechanical strength and high thermal conductivity. To address these limitations, SWCNT-based composite films were fabricated by combining SWCNTs with varying amounts of an inorganic-blended acrylic emulsion additive. The resulting SWCNT-based composite films exhibited significantly improved mechanical properties, with breaking strain and tensile strength values approximately thirty and two times higher, respectively, than those of the additive-free SWCNT film. Thermal conductivity decreased from 7.3 W/(m·K) for the additive-free SWCNT film to 2.1 W/(m·K) for the SWCNT-based composite films. Two types of TEGs were fabricated using the composite films: (1) the water-floating TEG, which generated a temperature difference through evaporative cooling; and (2) the standard TEG, which generated a temperature difference when vertically mounted on a heater. The output voltage of the first type of TEGs decreased as the additive amount increased, owing to reduced evaporative cooling. However, the second type of TEGs increased the output voltage by adding the appropriate amount of additive owing to the film’s low thermal conductivity. These findings are significantly helpful in using TEGs with appropriate designs and placements.

## 1. Introduction

In recent years, increasing global energy demand and escalating environmental issues, such as global warming, have driven a worldwide movement toward the realization of a sustainable energy society. Concurrently, advances in the Internet of Things (IoT) have facilitated the widespread deployment of sensors across diverse domains, including infrastructure, industry, medicine, and agriculture [1,2,3,4,5,6,7,8,9]. This progress has accelerated the emergence of a “smart society,” in which vast quantities of data are collected and shared in real time. The number of these sensors is expected to reach the trillions, underscoring their essential role in supporting and advancing the evolution of modern society [10,11,12].

However, as sensors become increasingly diverse and widely distributed, providing a stable power supply to each device has emerged as a significant challenge. Conventional chemical power sources, such as lithium-ion batteries, present several limitations, including finite lifespans, the need for periodic replacement, and environmental concerns associated with disposal. These drawbacks render them unsuitable for sensor systems that require long-term operation. Consequently, there is a substantial demand for energy-autonomous power sources that operate independently of chemical energy storage. In this context, energy harvesting technologies, which capture small amounts of ambient energy and convert them into electrical power, have attracted considerable attention [13,14,15,16,17]. Among energy harvesting technologies, thermoelectric power generation is regarded as a promising maintenance-free power supply solution because it directly converts thermal energy into electricity and does not depend on light or mechanical vibrations [18,19,20,21,22,23,24]. Moreover, it is capable of generating electrical power even from small temperature differences [25,26,27,28,29].

Under these circumstances, single-walled carbon nanotubes (SWCNTs) have attracted considerable attention as a next-generation flexible thermoelectric material owing to their advantages of flexibility, high electrical conductivity, and superior thermoelectric performance near 300 K [30,31,32,33,34,35,36]. However, despite the excellent mechanical properties of individual SWCNTs [37,38,39], the mechanical strength of SWCNT films is significantly reduced because they consist of aggregates of individual nanotubes held together primarily by van der Waals forces [40,41]. Additionally, although SWCNT films exhibit considerably lower thermal conductivity than isolated SWCNTs, their thermal conductivity remains higher than that of conventional inorganic thermoelectric materials, such as bismuth telluride alloys [42,43,44,45,46,47,48]. This elevated thermal conductivity presents a challenge in achieving a large temperature gradient within the film.

Recent studies have addressed these challenges by adjusting thermal conductivity and improving thermoelectric performance through control of the type and structure of SWCNTs, as well as by improving the mechanical properties of SWCNT thin films [49,50,51,52,53,54,55]. Some studies have investigated the enhancement of electrical conductivity and the Seebeck coefficient by combining SWCNTs with the conductive polymer polypyrrole [56]. With respect to mechanical properties, Zeng et al. reported achieving high mechanical strength alongside high thermal conductivity through orientation [57]. Furthermore, Kato et al. employed a blade coating method to fabricate SWCNT thin films with highly oriented, uniform structures and excellent mechanical properties [58]. Nevertheless, most of these studies have focused on improving either thermal conductivity, thermoelectric performance, or mechanical properties, with few evaluating multiple properties simultaneously.

In this study, to improve the high mechanical strength and stretchability of thermoelectric generators (TEGs) using SWCNTs, while maintaining the performance of the TEGs, the SWCNT-based composite films were prepared using an inorganic-blended acrylic emulsion as an additive. The resulting films exhibited high mechanical strength and stretchability, along with low thermal conductivity. With an additive amount of 20 mL, the composite film exhibited a tensile strength of 20.5 MPa, a breaking strain of 103%, and a thermal conductivity of 2.1 W/(m·K). Compared with the additive-free SWCNT film, this composite film showed a twofold increase in tensile strength, a thirtyfold increase in breaking strain, and a 70% reduction in thermal conductivity. The highest dimensionless figure of merit at 300 K was 5.0 × 10^−4^, achieved with an additive amount of 5 mL. Sequentially, two types of TEGs were fabricated using SWCNT-based composite films. The first type of TEGs was designed to float on water and does not require an external heat source [59,60], whereas the second type was intended to be placed vertically on a heat source [61,62]. The incorporation of an inorganic-blended acrylic emulsion enhanced the performance of the first type of TEG, but had little effect on the second type. Therefore, this study demonstrated the fabrication of SWCNT-based composite films with high mechanical strength, stretchability, and thermoelectric properties. Furthermore, the TEGs with high performance were demonstrated using these films. These findings provide insights into improving the thermoelectric properties of SWCNT-based composite films and inform the design of TEGs for specific applications.

## 2. Materials and Methods

**Figure 1:**
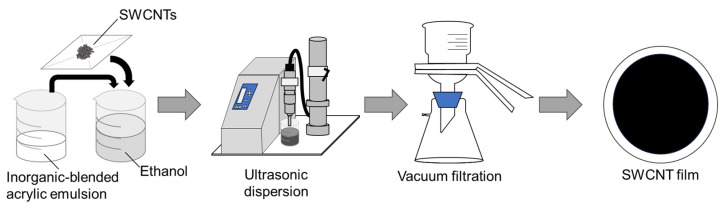
Fabrication process of SWCNT-based composite films.

Figure 1 illustrates the fabrication process of the SWCNT-based composite films. The materials and chemicals used in this study included SWCNT powders (ZEONANO SG101, ZEON, Tokyo, Japan), synthesized via the super-growth method [63], inorganic-blended acrylic emulsion (Kiwami NB spray, Make3, Osaka, Japan), and ethanol (Fujifilm Wako, Osaka, Japan). During preparation, the materials were mixed in a beaker, with the amount of inorganic-blended acrylic emulsion ranging from 0 to 20 mL, while the amounts of SWCNT powder and ethanol were maintained at 0.1 g and 50 mL, respectively. To prepare a uniform SWCNT composite dispersion, an ultrasonic homogenizer (Branson Sonifier SFX 250, Emerson, St. Louis, MO, USA) was used at 60% amplitude (nominal power of 200 W) for 30 min. The ultrasonic amplitude and frequency were 90 µm and 20 kHz, respectively, with a horn tip diameter of 12.7 mm. The rheology of composite SWCNT inks was examined using a rotational rheometer (MCR102e, Anton Paar, Graz, Austria) with a cone-plate geometry (Ø 50 mm) and a gap size of 0.5 mm, over a shear rate range of 0.1–1000 (1/s). The measurements were conducted at a constant temperature (293 K) controlled by a Peltier system. SWCNT-based composite films were subsequently fabricated via vacuum filtration. The SWCNT composite dispersion was deposited onto a 90 mm PETE membrane filter (ADVANTEC, Tokyo, Japan) positioned in a suction bottle. The filtration was conducted under a suction pressure of 0.08 MPa for approximately 1 h.

The microstructures of the SWCNT-based composite films were examined using field-emission scanning electron microscopy (FE-SEM; S-4800, Hitachi, Tokyo, Japan). Elemental mapping of the films was performed using FE-SEM equipped with electron backscattering diffraction (JSM-7100F, JEOL, Akishima, Japan). The crystal structures of the additives and composite films were analyzed by X-ray diffraction (XRD; Mini Flex II, Rigaku, Akishima, Japan) using Cu–Kα radiation (λ = 0.154 nm) over a 2*θ* range of 7–80°. Film thickness was measured using a digital micrometer (DM025, AsOne, Osaka, Japan), and the composite film density was determined from its mass and volume. The specific heat of the films was determined using differential scanning calorimetry (DSC-60PLUS, Shimadzu, Kyoto, Japan). The tensile strength and breaking strain of the SWCNT-based composite films were measured at approximately 300 K using a tensile testing machine (MX-1000N-FA, IMADA, Toyohashi, Japan). Test specimens were cut from the 90-mm-diameter composite films to dimensions of 22 mm (length) × 2.5 mm (width). To ensure measurement accuracy and reproducibility, five specimens from the same sample were tested. The wettability of the SWCNT-based composite films was determined using a drop shape analyzer (DSA100, Krüss GmbH, Hamburg, Germany) at approximately 300 K via the static sessile drop method. A water droplet of approximately 1 µL was placed on the film surface, and side-view images of the droplet were captured at 10 s intervals using a CCD camera. The contact angle of each droplet was then determined from the captured images.

All thermoelectric properties of the SWCNT-based composite films were measured in the in-plane direction at approximately 300 K. The Seebeck coefficient was determined at approximately 300 K using a custom-built measurement system with an accuracy of ±5%. One end of the film was attached to a heat sink, and the other end was connected to a Peltier module (FPH1-12704AC, Z-MAX, Tokyo, Japan). Two K-type thermocouples with a diameter of 0.1 mm were placed in contact with the center region of the thin film. The temperature difference between the thermocouples was varied from 0 to 4 K by controlling the current applied to the Peltier module using a DC power supply (PAB32-2, Kikusui, Yokohama, Japan), and the corresponding thermoelectric voltage was recorded at 1 K intervals. The Seebeck coefficient was determined from the slope of the thermoelectric voltage. The thermal diffusivity was measured with a precision of ±5% using a non-contact laser-spot periodic-heating radiation calorimetry method (TA33 Wave Analyzer, Bethel, Ishioka, Japan). The electrical conductivity was measured using a four-probe resistance meter (RT-70V, Napson, Tokyo, Japan) with an accuracy of ±3%. The thermal conductivity (*κ*) was calculated using the equation: *κ* = *αρC*, where *α*, *ρ*, and *C* represent thermal diffusivity, mass density, and specific heat, respectively. The power factor (*PF*) and dimensionless figure of merit (*ZT*) were obtained from the following equations: *PF* = *σS*^2^ and *ZT* = *σS*^2^*T*/*κ*, where *σ* and *S* denote the electrical conductivity and Seebeck coefficient, respectively.

## 3. Results and Discussion

### 3.1. Structural and Mechanical Properties of SWCNT-Based Composite Films

**Figure 2:**
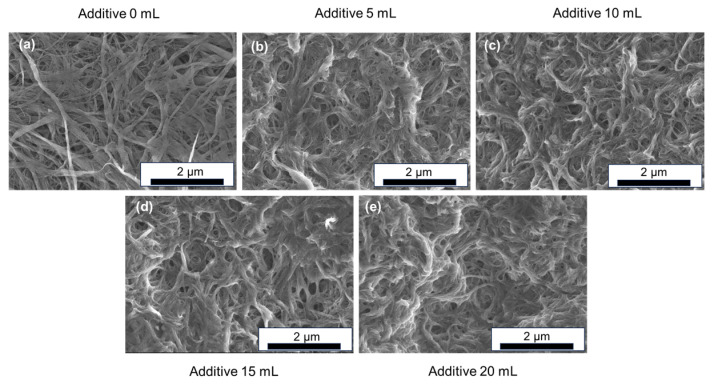
SEM images of (**a**) additive-free SWCNT film and SWCNT-based composite films with additive amounts of (**b**) 5 mL, (**c**) 10 mL, (**d**) 15 mL, and (**e**) 20 mL.

The microstructures of the SWCNT-based composite films containing various amounts of additive, as observed by FE-SEM, are presented in Figure 2. The SEM image of the additive-free SWCNT film is shown in Figure 2a. The SWCNT bundles exhibited a diameter ranging from several tens of nanometers to submicron scales. Considering that the diameter of an individual SWCNT used in this study is approximately 3–5 nm, even the thinnest bundles consisted of multiple nanotubes. The bundles showed no preferential alignment; rather, they appeared curved and entangled, forming voids and gaps within the network. The microstructures of the composite films incorporating 5, 10, 15, and 20 mL of additive are shown in Figure 2b, Figure 2c, Figure 2d, and Figure 2e, respectively. The SWCNT bundles and additives were not phase-separated; rather, the additives adhered to the surfaces of the SWCNT bundles. As the additive content increased, the gaps between the bundles became progressively smaller. Elemental mapping of the SWCNT-based composite films is shown in the Supplementary Materials (Figure S1). The analysis confirmed the presence of carbon originating from both the SWCNTs and the acrylic polymer. Additionally, calcium derived from calcium nitrate was detected and found to be uniformly distributed throughout the films, as further supported by XRD analysis provided in the Supplementary Materials (Figure S2) [64]. The XRD patterns of the composite films are shown in the Supplementary Materials (Figure S3).

**Figure 3:**
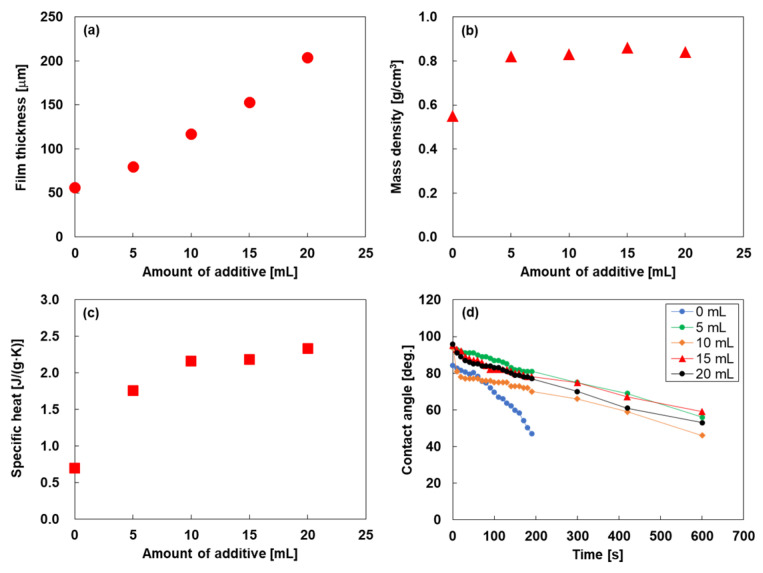
(**a**) Film thickness, (**b**) mass density, (**c**) specific heat, and (**d**) wettability of SWCNT-based composite films as functions of additive amount.

Figure 3 shows the relationship between the amount of additive and the film thickness, mass density, specific heat, and wettability of the SWCNT-based composite films. As shown in Figure 3a, the thickness of the additive-free SWCNT film was 56 μm, and it increased almost linearly with increasing additive content. The composite film containing 20 mL of additive exhibited the maximum thickness of 204 µm. As shown in Figure 3b, the mass density of the additive-free SWCNT film was 0.55 g/cm^3^. The addition of 5 mL of additive increased the mass density to 0.82 g/cm^3^, after which it remained nearly constant despite further increases in additive content. As shown in Figure 3c, the specific heat of the additive-free SWCNT film was 0.70 J/(g∙K). The specific heat increased to 2.2 J/(g∙K) when 10 mL of additive was introduced and remained approximately constant with further increases in additive content. The time dependence of the water contact angle for the films is shown in Figure 3d. The initial contact angles of all films ranged between 80° and 100°. However, the contact angle of the additive-free SWCNT film decreased rapidly over time, while those of the composite films decreased more gradually and at nearly constant rates, irrespective of the additive amount. This behavior can be attributed to the higher hydrophobicity of the acrylic polymer compared to that of the SWCNTs.

**Figure 4:**
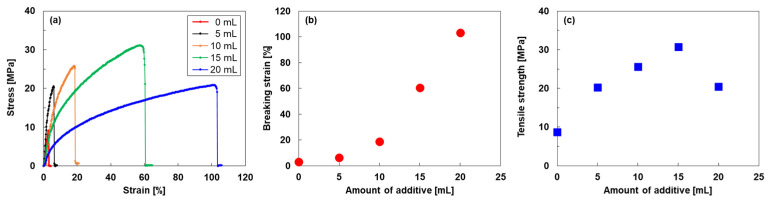
(**a**) Stress–strain curves of SWCNT-based composite films, (**b**) breaking strain and (**c**) tensile stress as functions of additive amount.

Figure 4 shows the results of the tensile tests of the SWCNT-based composite films. As shown in Figure 4a, the stress–strain curves of the SWCNT-based composite films varied significantly depending on the amount of additive. The additive-free SWCNT film exhibited the lowest breaking strain and tensile strength because the SWCNT bundles were connected only by point contacts through van der Waals forces. In contrast, the films containing additives exhibited greater breaking strain and tensile stress than the additive-free SWCNT film. As the amount of additive increased, the deformation region of the stress–strain curves expanded, and the breaking strain increased. This indicates that films containing larger amounts of additives possessed higher strength and stretchability because the acrylic polymer additives acted as binders. For quantitative analysis, the dependencies of the breaking strain and tensile stress on the amount of additive are shown in Figure 4b and Figure 4c, respectively. The breaking strain increased quadratically with increasing additive content. The SWCNT-based composite film containing 20 mL of additive exhibited a breaking strain of 103%, which was 34 times higher than that of the additive-free SWCNT film. In contrast, the tensile stress increased linearly with the additive amount up to 15 mL, at which point it reached the maximum value of 30.7 MPa, which was 3.5 times higher than that of the additive-free SWCNT film. Upon further increasing the additive amount, the tensile strength decreased to 20.5 MPa. This phenomenon occurred because excessive additives formed pathways that bypassed the SWCNT bundles, leaving only the acrylic polymer. Consequently, the stretchability of the acrylic polymer became the dominant factor.

### 3.2. Thermoelectric Properties of SWCNT-Based Composite Films

**Figure 5:**
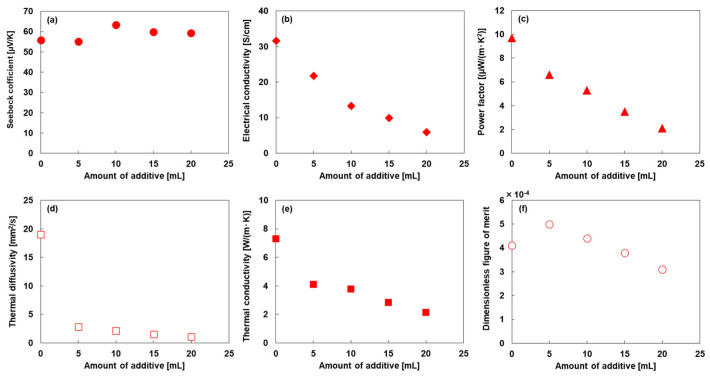
(**a**) Seebeck coefficient, (**b**) electrical conductivity, (**c**) power factor, (**d**) thermal diffusivity, (**e**) thermal conductivity, and (**f**) dimensionless figure of merit of SWCNT-based composite films as functions of additive amount.

Figure 5 presents the in-plane thermoelectric properties of the SWCNT-based composite films with varying amounts of additive, measured at approximately 300 K. As shown in Figure 5a, the Seebeck coefficient of the additive-free SWCNT film was 55.8 μV/K, indicating that the film exhibited *p*-type semiconducting behavior. Even with increasing additive content, the Seebeck coefficient changed very little. The Seebeck coefficient (*S*) is expressed using Equation (1): [65]

(1) $$S=\frac{8\pi^{2}k_{B}^{2}T}{3eh^{2}}m^{*}{\left(\frac{\pi }{3n}\right)}^{\frac{2}{3}}\left(1+R\right),$$

where m*, *n*, and *R* represent the effective mass, carrier concentration, and scattering factor, respectively. The Seebeck coefficient is inversely proportional to the two-thirds power of the carrier concentration when the effective mass is fixed. Therefore, it can be concluded that the carrier concentration did not significantly change with increasing additive content. As shown in Figure 5b, the electrical conductivity decreased with increasing additive amount. The electrical conductivity (*σ*) is expressed by Equation (2): [66]

(2) σ=qnμ,

where *q* and *μ* denote the elementary charge and carrier mobility, respectively. As the amount of additives does not significantly affect the carrier concentration, the primary cause of the reduced electrical conductivity is the decrease in carrier mobility. This reduction occurs because the calcium nitrate-based inorganic materials and acrylic polymers hinder carrier flow within the composite films. As shown in Supplementary Materials (Figure S4), the viscosity of the SWCNT composite dispersion supported this phenomenon. As the additive amount increased, the viscosity decreased at lower shear rates, suggesting that the additives entered the gaps within the SWCNT bundles. As shown in Figure 5c, the relationship between additive amount and power factor is similar to that between additive amount and electrical conductivity. At an additive amount of 20 mL, the composite film exhibited a power factor of 2.1 μW/(m·K^2^), which is approximately 80% lower than that of the additive-free SWCNT film. As shown in Figure 5d, the thermal diffusivity of the additive-free SWCNT film was 19.0 mm^2^/s. When 5 mL of the additive was incorporated into the film, the thermal diffusivity rapidly decreased to 2.9 mm^2^/s. With further increases in additive content, the thermal diffusivity gradually decreased, reaching 1.1 mm^2^/s at 20 mL of additive. As shown in Figure 5e, the relationship between thermal conductivity and additive amount is similar to that between thermal diffusivity and additive amount. However, the rate of decrease in thermal conductivity upon the addition of the additive was lower than that observed for thermal diffusivity. This is because both the mass density and specific heat increased with increasing additive amount, as shown in Figure 3b and Figure 3c, respectively. The highest thermal conductivity of 7.3 W/(m·K) was observed for the additive-free SWCNT film, while the lowest thermal conductivity of 2.1 W/(m·K) was obtained for the composite film containing 20 mL of additive. The inorganic-blended acrylic emulsion primarily consisted of calcium nitrate and an acrylic polymer. The thermal conductivities of calcium nitrate and acrylic polymer are 0.57 and 0.21 W/(m·K), respectively [64,67], both of which are lower than that of the additive-free SWCNT film. Therefore, as shown in Figure 5f, the thermal conductivity of the SWCNT-based composite films decreased with increasing additive content. When 5 mL of additive was included in the film, the dimensionless figure of merit reached its highest value of 5.0 × 10^−4^, which was approximately 20% higher than that of the additive-free SWCNT film. This trend differs from that of the power factor because the reduction in thermal conductivity has a greater influence than the decrease in power factor at an additive amount of 5 mL. The SWCNT-based composite film containing 15 mL of additive exhibited a dimensionless figure of merit of 3.8 × 10^−4^, which was comparable to that of the additive-free SWCNT film. Therefore, by incorporating the inorganic-blended acrylic emulsion, films with high mechanical strength and stretchability were obtained while maintaining a dimensionless figure of merit comparable to that of the additive-free SWCNT film.

### 3.3. Performance of TEGs Using SWCNT-Based Composite Films

**Figure 6:**
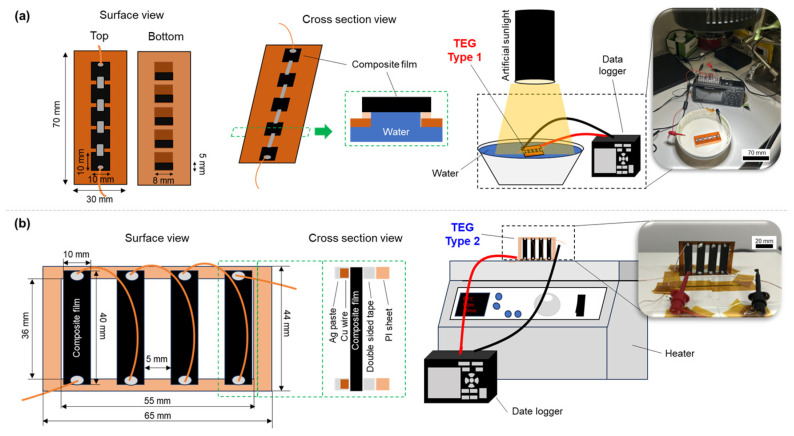
(**a**) Configuration and measurement setup of the water-floating TEG, and (**b**) configuration and measurement setup of the standard TEG vertically mounted on a heater.

To evaluate the effectiveness of the SWCNT-based composite films, two types of TEGs were fabricated using the composite films, and their performance was measured, as shown in Figure 6. The fabrication process and measurement procedure of the first type of TEG, referred to as the water-floating TEG, are shown in Figure 6a. The detailed fabrication process is described in our previous report [60]. In brief, the SWCNT-based composite film was cut into five pieces measuring 10 mm in length and 10 mm in width. The substrate was a 70 mm × 30 mm polyimide sheet (Kapton, DuPont, Wilmington, DE, USA) with five rectangular openings (5 mm × 8 mm) arranged in a straight line. The five cut composite films were attached to their respective openings on the polyimide substrate using double-sided tape. This configuration allowed the composite films to come into direct contact with water and generate a temperature difference through evaporative cooling. The five composite films were connected in series using fine copper wires and silver paste, with two additional fine copper wires attached at both ends to measure the output voltage. The water-floating TEGs were irradiated with artificial sunlight at an intensity of approximately 1000 W/m^2^ (SOLAX 100 W XC-100 B, SERIC, Koshigaya, Japan), while the water temperature was maintained at 35 °C. The output voltages and temperatures of the water-floating TEGs were recorded for 30 min after light exposure using a data logger (GL240, GRAPHTEC, Yokohama, Japan). Figure 6b shows the fabrication process and measurement procedure of the second type of TEG, namely, the standard TEGs vertically mounted on a heater. The SWCNT-based composite film was cut into four 40 mm × 10 mm pieces. The substrate was a 65 mm × 44 mm polyimide sheet (Kapton, DuPont, Wilmington, DE, USA) with a 55 mm × 36 mm rectangular opening to minimize contact with the composite films. The composite films were spaced 5 mm apart and attached to the substrate using double-sided tape at both ends. The four composite films were connected with thin copper wires and silver paste along the top and bottom edges of adjacent films. Two additional thin copper wires were attached to each end of the films to measure the output voltage. The TEG was positioned perpendicular to the heater (DP-1L, AsOne, Osaka, Japan) to establish a temperature difference between the top and bottom surfaces of the composite films. The heater temperature was maintained at 60 °C, and the output voltage was recorded for 15 min using a data logger (GL240, GRAPHTEC, Yokohama, Japan).

**Figure 7:**
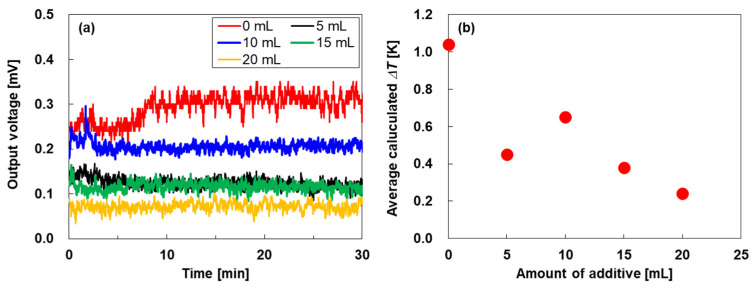
(**a**) Time dependence of output voltage of water-floating TEGs with various additive amounts under artificial sunlight irradiation, and (**b**) calculated temperature difference in TEGs as a function of additive amount.

Figure 7 shows the performance of the water-floating TEGs fabricated using the SWCNT-based composite films. The time dependence of the output voltage generated by the water-floating TEGs is presented in Figure 7a. Regardless of the amount of additive, all TEGs produced a stable voltage output. The highest output voltage was observed with the TEG incorporating additive-free SWCNT films, averaging approximately 0.3 mV. When additives were incorporated into the SWCNT films, the generated voltages were lower than those of the TEG using the additive-free SWCNT films. In particular, the TEG with composite films containing 20 mL of additive exhibited the lowest output voltage of approximately 0.07 mV, corresponding to one-fourth of that of the TEG with the additive-free SWCNT films. To evaluate TEG performance, the temperature difference across the TEG films was calculated from the measured Seebeck coefficient (Figure 5a) and output voltage (Figure 7a), as shown in Figure 7b. The temperature difference generated in the additive-free SWCNT films was 1.04 K and decreased with increasing additive content. The reduction in the performance of the water-floating TEGs upon the introduction of additives occurred because evaporative cooling was suppressed owing to insufficient capillary action of water through the composite films. The capillary action was hindered by the high hydrophobicity of the acrylic polymer and the filling of the gaps between the SWCNT bundles by the additive.

**Figure 8:**
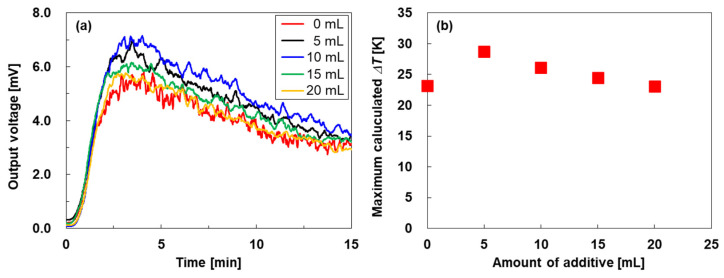
(**a**) Time dependence of output voltage of standard TEGs vertically mounted on a heater with various additive amounts, and (**b**) calculated temperature difference in TEGs as a function of additive amount.

Figure 8 shows the performance of the standard TEGs fabricated using the SWCNT-based composite films vertically mounted on a heater. The time dependence of the output voltage generated by the TEGs is shown in Figure 8a. The output voltages of all TEGs increased during heating and reached their maximum values at approximately 3.5 min. When the heating time was further extended while maintaining the heater temperature at 60 °C, the output voltages of all TEGs gradually decreased because heat was transferred from the bottom to the top, thereby reducing the temperature difference. When comparing the maximum output voltages of all TEGs, the highest output voltage of 7.0 mV was observed for the TEG with composite films containing 10 mL of additive. Conversely, the lowest output voltage of 5.5 mV was obtained with the TEG using additive-free SWCNT films. Figure 8b shows the calculated temperature difference across the TEG films, based on the measured Seebeck coefficient (Figure 5a) and maximum output voltage (Figure 8a). The temperature difference generated in the additive-free SWCNT films was 23.2 K and increased to 28.7 K in the TEG with composite films containing 5 mL of additive. With further increases in additive content, the temperature difference decreased linearly. When the additive amount reached 20 mL, the temperature difference in the TEG was nearly the same as that of the TEG with the additive-free SWCNT films. Therefore, the TEG with composite films containing 5 mL of additive exhibited the highest output voltage and temperature difference, even though the composite film with 20 mL of additive showed the lowest thermal conductivity, as shown in Figure 5e. This phenomenon can be explained by the following mechanism. When the additive amount is high (20 mL), the gaps between the SWCNT bundles are filled with additive components, which reduce heat dissipation in the films and consequently decrease the temperature difference. Conversely, when the additive amount is low (5 mL), the thermal conductivity is lower than that of the additive-free SWCNT films, while the gaps between the SWCNT bundles remain, allowing efficient heat dissipation to be maintained. Consequently, the temperature difference within the film increases, resulting in a maximized output voltage. Although the output voltages of the TEGs with SWCNT-based composite films containing higher additive amounts (15 and 20 mL) were not the highest, they were comparable to those of the TEG with the additive-free SWCNT films. Therefore, by incorporating an inorganic-blended acrylic emulsion, we demonstrated that TEGs vertically mounted on a heater can achieve high mechanical strength and stretchability while maintaining performance equivalent to that of the additive-free SWCNT films.

## 4. Conclusions

To achieve high mechanical strength, stretchability, and low thermal conductivity, SWCNT-based composite films were fabricated by combining SWCNTs with varying amounts of an inorganic-blended acrylic emulsion additive. The additive components adhered to the surface of the SWCNT bundles, and the gaps between the bundles decreased as the additive content increased. High mechanical strength and stretchability were achieved in the SWCNT-based composite film containing 20 mL of additive, with a breaking strain and tensile strength of 103% and 20.5 MPa, respectively. In addition, this composite film exhibited the lowest thermal conductivity of 2.1 W/(m·K), which was approximately 70% lower than that of the additive-free SWCNT film. As the dimensionless figure of merit of the composite films was comparable to that of the additive-free SWCNT film, SWCNT-based composite films with high mechanical strength, stretchability, and low thermal conductivity were successfully achieved without compromising thermoelectric performance. To investigate the effectiveness of the SWCNT-based composite films, two types of TEGs were fabricated. The first type was the water-floating TEGs, which generated a temperature difference through evaporative cooling. The output voltage decreased with increasing additive content due to reduced evaporative cooling, as the gaps between the bundles were filled with additive components. The second type was the standard TEGs, which generated a temperature difference when vertically mounted on a heater. In this case, the output voltage increased when an appropriate amount of additive was added, owing to the composite film’s low thermal conductivity and high heat dissipation. These findings provide valuable insights for designing and deploying TEGs with optimized configurations and application environments.

## Data Availability

The data presented in this study is available on request from the corresponding author.

## Supplementary Materials

The following supporting information can be downloaded at: https://www.mdpi.com/article/10.3390/nano15231817/s1. Figure S1: SEM images and elemental mapping of SWCNT-based composite films prepared with different additive volumes: (a–c) 5 mL, (d–f) 10 mL, (g–i) 15 mL, and (j–l) 20 mL. Panels show (a,d,g,j) SEM images, (b,e,h,k) carbon mapping, and (c,f,i,l) calcium mappings. Figure S2: XRD pattern of the dried inorganic-blended acrylic emulsion. Figure S3: The XRD patterns of the composite films. Figure S4: Viscosity of SWCNT composite dispersion as a function of shear rate.
