# Supplementary material for: SWCNT-Based Composite Films with High Mechanical Strength and Stretchability by Combining Inorganic-Blended Acrylic Emulsion for Various Thermoelectric Generators
Source: Nanomaterials (Basel). 2025 Dec 1;15(23):1817. doi: 10.3390/nano15231817 (PMC12693934; doi:10.3390/nano15231817)
Supplement: Supplementary file 1 [file nanomaterials-15-01817-s001.zip › nanomaterials-4016447-supplementary.pdf]

nanomaterials

SWCNT-Based Composite Films with High Mechanical Strength and Stretchability by Combining Inorganic-Blended Acrylic Emulsion for Various Thermoelectric Generators

Yuto Nakazawa [1], Yoshiyuki Shinozaki [1], Hiroto Nakayama [1], Shuya Ochiai [1], Shugo Miyake [2] and Masayuki Takashiri [1,*]

[1] Department of Materials Science, Tokai University, Hiratsuka 259-1292, Kanagawa, Japan
[2] Department of Mechanical Engineering, Setsunan University, Neyagawa 572-8508, Osaka, Japan
* Correspondence: takashiri@tokai.ac.jp

Supplemental information

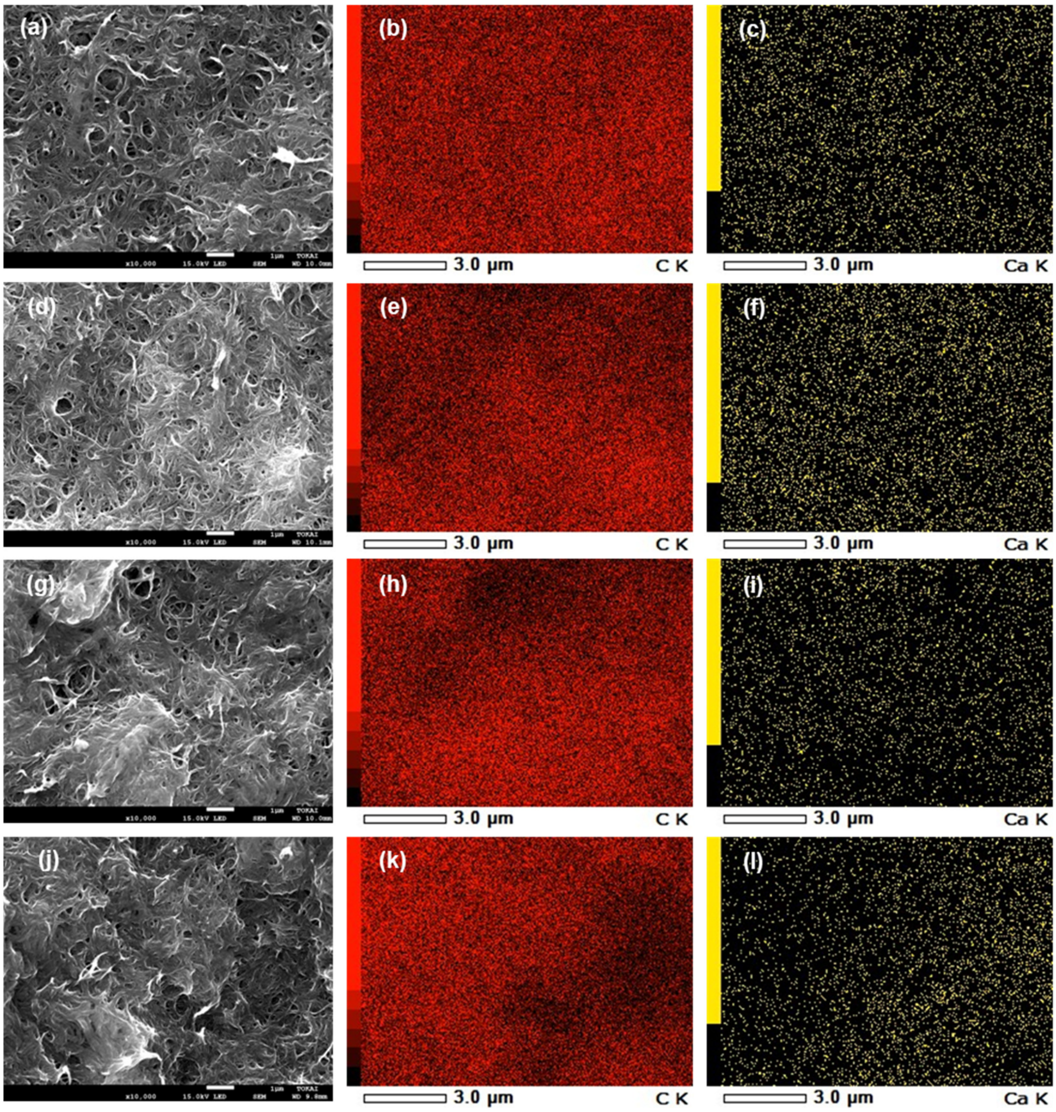

Figure S1. SEM images and elemental mapping of SWCNT-based composite films prepared with different additive volumes: (a–c) 5 mL, (d–f) 10 mL, (g–i) 15 mL, and (j–l) 20 mL. Panels show (a, d, g, j) SEM images, (b, e, h, k) carbon mapping, and (c, f, i, l) calcium mappings.

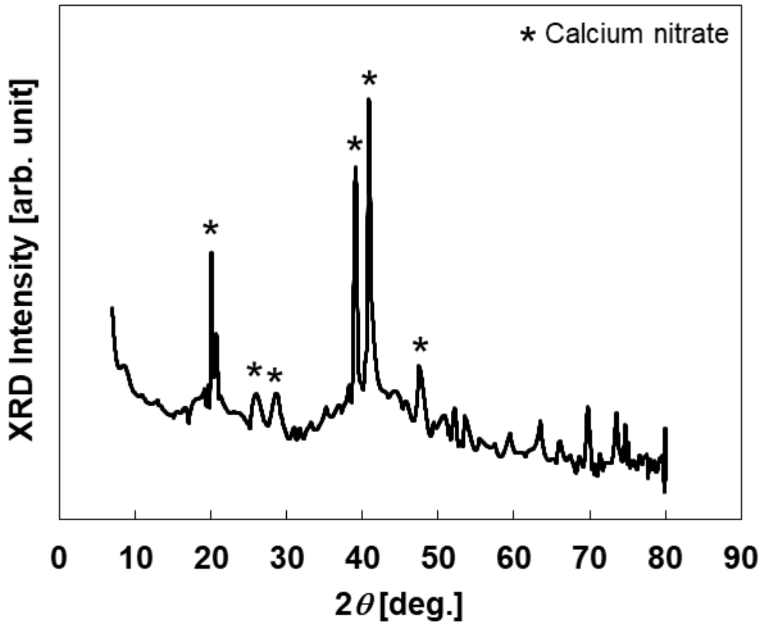

Figure S2. XRD pattern of the dried inorganic-blended acrylic emulsion.

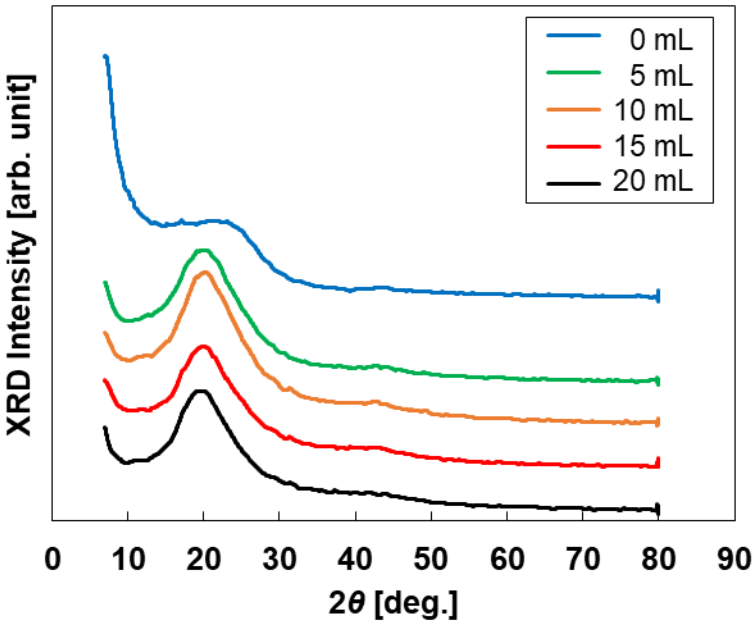

Figure S3. The XRD patterns of the composite films.

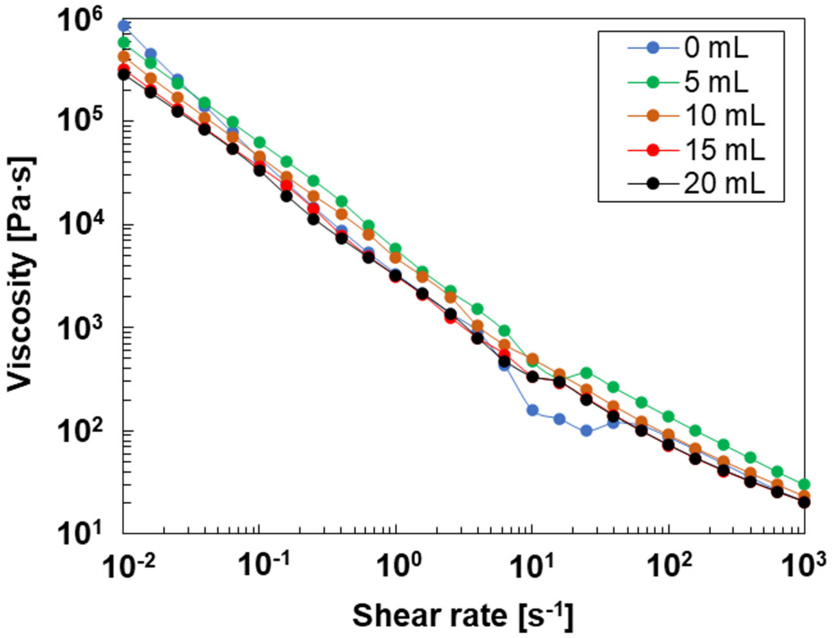

Figure S4. Viscosity of SWCNT composite dispersion as a function of shear rate.
